# Frequency and Clinicopathological Characteristics of Patients With *KRAS/BRAF* Double-Mutant Colorectal Cancer: An *In Silico* Study

**DOI:** 10.3389/pore.2022.1610206

**Published:** 2022-02-24

**Authors:** Shiro Uchida, Takaaki Kojima, Takashi Sugino

**Affiliations:** ^1^ Division of Diagnostic Pathology, Kikuna Memorial Hospital, Yokohama, Japan; ^2^ Pathology Division, Shizuoka Cancer Center, Shizuoka, Japan; ^3^ Department of Human Pathology, Juntendo University School of Medicine, Tokyo, Japan; ^4^ Graduate School of Bioagricultural Sciences, Nagoya University, Nagoya, Japan

**Keywords:** cancer, *KRAS*, *BRAF*, bioinformatic analysis, colon, double mutation

## Abstract

*KRAS* and *BRAF* mutations are currently thought to be mutually exclusive as their co-occurrence is extremely rare. Therefore, clinicopathological and molecular characteristics of colorectal carcinoma with *KRAS/BRAF* double mutations are unclear. We aimed to investigate the frequency and clinicopathological characteristics of double-mutant colorectal carcinoma and its differences from *KRAS/BRAF* single-mutant colorectal carcinoma using bioinformatics tools. We estimated the *KRAS/BRAF* double mutation frequency in the whole exon and coding sequences via bioinformatic analyses of three datasets from cBioPortal. We compared the clinicopathological characteristics, microsatellite instability status, *BRAF* classification, and tumor mutation burden of patients harboring the double mutants with those of patients harboring *KRAS* or *BRAF* single mutations. We integrated three large datasets and found that the frequency of the *KRAS/BRAF* double mutation in the dataset was 1.2% (29/2347). The double mutation occurred more frequently in males, with a slightly higher occurrence in the right side of the colon. Sex, histological type, histological grade, microsatellite instability, and tumor mutation burden of the patients harboring *KRAS*-mutant, *BRAF*-mutant, and double-mutant colorectal carcinoma varied significantly. The frequency of double-mutant colorectal carcinoma was 60 times higher than that previously reported. Significantly fewer double-mutant colorectal carcinoma cases were classified as *BRAF* class 1 and more were classified as unknown. Our findings indicate that the biological characteristics of double-mutant tumors are different from those of single-mutant tumors.

## Introduction

Colorectal cancer (CRC) is the second-most common cancer in women and the third-most common cancer in men [1]. CRC progresses through several steps associated with specific genetic and epigenetic alterations in various oncogenes and tumor suppressor genes [2]. Kirsten rat sarcoma viral oncogene homolog (*KRAS*) and v-RAF murine sarcoma viral oncogene homolog B1 (*BRAF*) are the major oncogenic drivers of CRC [3]. Approximately 30–45% of patients with CRC harbor *KRAS* mutations and 5–20% harbor *BRAF* mutations [4]. *KRAS* and *BRAF* encode proteins involved in the Ras–Raf–MEK–ERK signaling pathway. *KRAS* can also activate other signaling pathways, such as the PIK3CA–AKT–mTOR pathway, which regulates protein translation and cell survival [5] Therefore, gain-of-function *KRAS* and *BRAF* mutations activate these pathways that act as molecular switches leading to cellular growth and proliferation and are associated with primary resistance to epidermal growth factor receptor (EGFR) inhibitors [6,7]. Recent studies have shown that *BRAF* V600-mutated CRC and *BRAF* non-V600-mutated CRC have different prognoses and different sensitivities to drugs; furthermore, the proposed *BRAF* mutations can be grouped into three classes (1, 2, and 3) [8,9] Currently, combinatorial therapy with cytotoxic chemotherapeutic agents and molecular targeted drugs (bevacizumab) are recommended as the first-line therapy for *KRAS/BRAF*-mutant CRC [10].

G12D, G12V, and G13D, the most common missense *KRAS* mutations and *BRAF* V600E have been recognized as being mutually exclusive [11,12]. In previous studies, the double *KRAS/BRAF* mutation frequency was 0.02% (1/4,170) [13–19]. However, reports regarding the occurrence of *KRAS* and *BRAF* double mutants have recently emerged [20–25]. To the best of our knowledge, only 11 cases have presented the co-occurrence of *KRAS* and *BRAF* mutations, indicating that this mutation is extremely rare. Owing to the rarity of the *KRAS/BRAF* double mutation, the clinicopathological and molecular characteristics of *KRAS/BRAF* double-mutant tumors and differences in the biology of *KRAS* or *BRAF* single-mutant CRC and *KRAS/BRAF* double-mutant CRC remain unknown.

In this study, we analyzed the frequency of *KRAS/BRAF* double mutations and the methods used for detecting these double mutations and determined the frequency of double-mutant CRC from three public datasets using bioinformatic tools. Additionally, we examined the clinicopathological features, microsatellite instability (MSI) status, tumor mutation burden (TMB), CpG island methylator phenotype (CIMP), *BRAF* classification, and the clinicopathological and molecular differences between CRCs with single *KRAS* or *BRAF* mutations and those with double mutations. To our knowledge, this is the first study to determine the *KRAS/BRAF* double mutation frequency in a large dataset. This study demonstrated the frequency of double-mutant colorectal carcinoma and clarified the clinicopathological and molecular features of double-mutant CRC.

## Materials and Methods

### Data Collection

Genomic and clinical data associated with tumor samples from patients with colorectal adenocarcinoma (The Cancer Genome Atlas [TCGA] PanCancer Atlas; n = 594), metastatic CRC [Memorial Sloan-Kettering Cancer Center (MSKCC), n = 1,134] [26], and colorectal adenocarcinoma [Dana-Farber Cancer Institute (DFCI), n = 619] [27] were accessed online via the cBioPortal. We extracted datasets for *KRAS* mutation, *BRAF* mutation, and KRAS/BRAF double mutation from all the samples (n = 2347), including TCGA, MSKCC, and DFCI tumor samples combined. Clinicopathological features, including age, sex, tumor location, histological type, grade (G1, G2, and G3), tumor–node–metastasis classification (only TCGA), stage, CIMP (only DFCI) and overall survival data were obtained from TCGA and MSKCC via the cBioPortal. Additionally, a list of amino-acid changes and information regarding the pathological significance of each *KRAS* or *BRAF* mutation were accessed using COSMIC [28]. Allele frequency was assessed using cBioPortal (Supplementary Table S1).

### Mutation Data

In TCGA, MutSig2CV was applied to quality-controlled mutation data to evaluate the significance of the mutated genes and estimate the mutation densities of samples. MutSig2CV [29] combines evidence from the background mutation rate, clustering of mutation on hotspots, and conservation of mutated sites to calculate false discovery rates (q-values). Genes with q-value <0.1 were considered significant [30].

In MSKCC, the thresholds on the coverage depth, number of mutant reads, and variant frequency for rejecting almost false-positive calls were determined. First-tier variants were filtered using the following criteria: coverage depth ≥20×, mutant reads ≥8, and variant frequency ≥2%. Second-tier variants were filtered according to the following criteria: coverage depth ≥20×, mutant reads ≥10, and variant frequency ≥5% [31].

In DFCI, C > T mutations consistent with a 20:1 single-strand bias were filtered out based on the read pair orientation to remove artifacts resulting from the hydrolytic deamination of cytosine to form uracil, specifically in formalin-fixed, paraffin-embedded samples. The MutSigCV suite of tools and manual curation was used to identify significantly mutated genes [27].

### Microsatellite Instability Analysis

For TCGA PanCancer Atlas and MSKCC samples, the microsatellite status was assessed via MSIsensor, a computational algorithm that analyses sequencing reads at designated microsatellite regions in tumor-normal pairs reporting the percentage of unstable loci as a cumulative score [32]. MSI sensor scores ≥10 were defined as MSI-high (MSI-H), scores ≥3 and <10 as MSI-intermediate (MSI-I), and scores <3 as microsatellite stable (MSS) [33]. For DFCI samples, microsatellite status was analyzed using 10 microsatellite markers (D2S123, D5S346, D17S250, BAT25, BAT26, BAT40, D18S55, D18S56, D18S67, and D18S487) as previously described [27].

### Estimation of TMB

TMB was estimated from TCGA PanCancer Atlas for *KRAS* mutation (n = 212), BRAF mutation (n = 57), and double mutation (n = 6) as the total number of mutations per sample/38 Mb. Furthermore, TMB was estimated from MSKCC for *KRAS* mutation (n = 470), *BRAF* mutation (n = 104), and double mutation (n = 17) as the total number of mutations per sample/1.22 Mb. The denominators 38 and 1.22 Mb represented the estimated length of human exome (38 Mb) reported in the TCGA database [34] and the estimated length of captured region (tumor DNA) of 468 cancer-related genes in the MSKCC database, respectively [35] The samples were classified as TMB-high if they had ≥12 mutations per megabase (mut/Mb), as previously described [36]. Additionally, the TMB of single-mutant and double-mutant CRC mutants from the two datasets were integrated (TCGA, MSKCC). Based on the integrated data hosted on TCGA and MSKCC, we compared the TMB in patients with KRAS-mutant (n = 682), BRAF-mutant (n = 161), and double-mutant (n = 23) tumors.

### *BRAF* Classification

Amino-acid changes in *BRAF* in single-mutant and double-mutant cases (TCGA, MSKCC, and DFCI) were classified into classes 1, 2, and 3 according to previous reports [8,9]. Amino-acid changes that did not belong to any of these classes were classified as unknown.

### Comparison of Clinicopathological Features, MSI Status, and TMB of CRC Mutants in TCGA, MSKCC, and DFCI Datasets

We integrated the clinicopathological information of the CRC mutants from the three datasets (TCGA, MSKCC, and DFCI) and performed a comparative analysis among *KRAS*-mutant, *BRAF*-mutant, and double-mutant CRCs. In the DFCI dataset, data on histological type and TMB were not available. Therefore, histological type and TMB were measured only in TCGA and MSKCC datasets. The histological type information was not available for the DFCI dataset; therefore, the percentage for histological type was calculated from 543 cases in *KRAS*-mutant CRC, 126 cases in *BRAF*-mutant CRC, and 18 cases in double-mutant CRC. MSI status was calculated only in the TCGA and MSKCC datasets because the evaluation method was different in the DFCI dataset. Conversely, information on CIMP was only available in the DFCI dataset. Instances of N/A were omitted from the percentage calculation.

### Statistical Analyses

The clinicopathological features of patients with *KRAS* and *BRAF* single-mutant and double-mutant CRC were analyzed using the chi-square and Fisher’s exact tests. Comparisons between the single mutation (*KRAS* or *BRAF*) and double mutations in hotspot and other mutation sites of *KRAS* and V600E and non-V600E mutations of *BRAF* were analyzed using the chi-square test. The TMB of patients with *KRAS* mutant, *BRAF* mutant, and double-mutant CRC was analyzed using the Mann–Whitney *U* test. The Bonferroni post-test correction was used to reduce the likelihood of false positives. Between-group comparisons (*KRAS* mutation vs. double mutation, *BRAF* mutation vs. double mutation) were performed, and *p <* 0.025 (0.05/2) was considered statistically significant. All statistical analyses were performed using R software, version 4.0.3 (R Foundation for Statistical Computing, Vienna, Austria).

## Results

### Comparison of the Frequency of the Double KRAS/BRAF Mutation Between the Present and Previous Studies

The data from previous reports and the present study are summarized in Tables 1, 2. In our study, the double *KRAS/BRAF* mutation frequency from the integrated analysis of TCGA, MSKCC, and DFCI data was 1.2% (29/2,347). The frequency was 1% (6/594) in TCGA, 1.5% (17/1,134) in MSKCC, and 1% (6/619) in DFCI data. Codons 12 (exon 2), 13 (exon 2), 59 (exon 3), 61 (exon 3), 117 (exon 4), and 146 (exon 4) are the hotspots of *KRAS* mutation [37]. Codon 600 (exon 15) is the hotspot of V600E and non-V600E *BRAF* mutations [3,37] The numbers of each of the three mutations (*KRAS* mutation, *BRAF* mutation, and double mutation) that occurred in the hotspots of codons 12, 13, 61, 117, and 146 in TCGA, MSKCC, and DFCI were determined.

**TABLE 1:** Frequency of *KRAS* mutation, *BRAF* mutation, and *KRAS*/*BRAF* double mutation and target sites reported in previous studies.

References	*KRAS* mut (%)	*BRAF* mut (%)	Double mut (%)	Sequence area
13	397/1,063 (37.4)	60/999 (6.9)	1/999 (0.1)	*KRAS* (codon 12,13)*BRAF* (V600E)
14	450/1,077 (41.8)	26/397 (6.5)	0/397 (0)	*KRAS* (codon 12, 13)*BRAF* (V600E)
15	90/315 (28.8)	33/315 (10.6)	0/315 (0)	*KRAS* (codon 12, 13)*BRAF* (V600E)
16	565/1,294 (43.7)	102/1,189 (8.5)	0/1,189 (0)	*KRAS* (codon 12, 13, 61)*BRAF* (codon 600)
17	63/200 (31.5)	14/200 (6.5)	0/200 (0)	*KRAS* (codon 12, 13)*BRAF* (codon 15, V600)
18	136/315 (43.2)	11/309 (3.6)	0/309 (0)	*KRAS* (codon 12, 13)*BRAF* (V600E)
19	299/747 (40.0)	36/761 (4.7)	0/761 (0)	*KRAS* (codon 12, 13, 61, 146)*BRAF* (V600E)
Total	2000/5,011 (39.9)	282/4,170 (6.8)	1/4,170 (0.02)	

Mut, mutation.

**TABLE 2:** Frequency of *KRAS* mutation, *BRAF* mutation, and *KRAS*/*BRAF* double mutation and the sequence area obtained from the integrated analysis of TCGA, MSKCC, and DFCI datasets in this study.

Data set	*KRAS* mut (%)	*BRAF* mut (%)	Double mut (%)	Method	Sequence area
TCGA	212/594 (35.7)	57/594 (9.6)	6/594 (1.0)	NGS	Whole exon
MSKCC	470/1,134 (41.4)	104/1,134 (9.2)	17/1,134 (1.5)	NGS	CDS of 468 genes, including *KRAS, BRAF*
DFCI	167/619 (27)	121/619 (19.5)	6/619 (1.0)	NGS	Whole exon
Total	849/2347 (36.1)	282/2347 (12)	29/2347 (1.2)		

CDS, coding sequence; mut, mutation; mut, mutation; NGS, next-generation sequence.

### Comparison of the Hotspots of Single and Double Mutations of KRAS and BRAF

**FIGURE 1:**
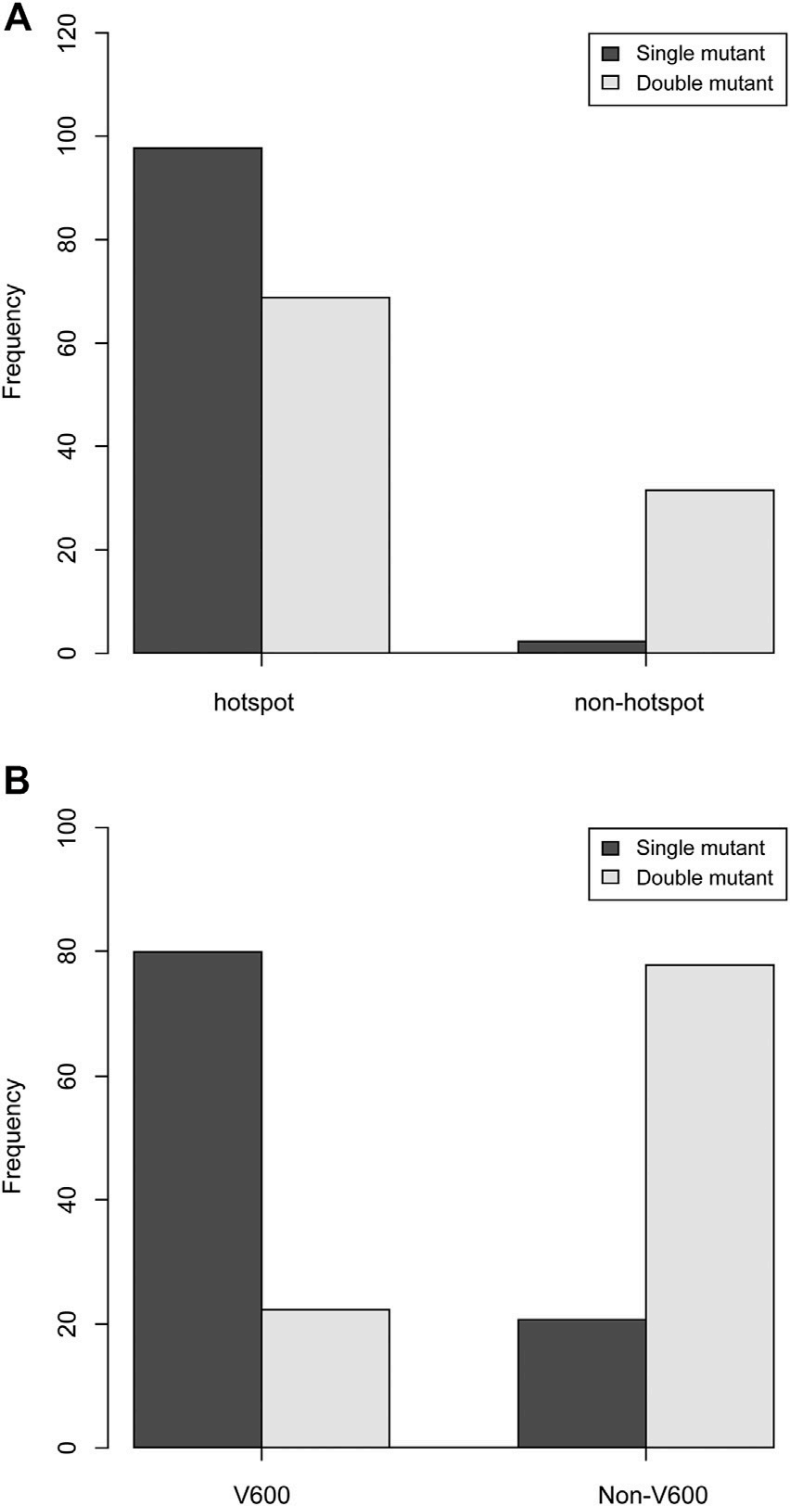
Comparison of hotspots of *KRAS* and *BRAF* single and double mutations. **(A)** Compared to single-mutant CRC, double-mutant CRC had significantly fewer hotspot mutations and had more non-hotspot mutations (*p* < 0.01). **(B)** Compared to single-mutant CRC, double-mutant CRC had significantly fewer V600 mutations and had more non-V600 mutations (*p* < 0.01).

Double-mutant CRC cases had significantly more non-hotspot and non-V600E mutations than single-mutant CRC cases (*p* < 0.01, respectively). The *KRAS* single mutation appeared in 97.8% hotspots, whereas the double mutation appeared in 68.8% hotspots and 31.3% other sites (Figure 1A). Moreover, 79.5% of the *BRAF* single mutations were of the V600E type, whereas 20.5% of them were of the non-V600E type. Although 22.2% of the *BRAF* mutations in double-mutant CRC were of the V600E type, 77.8% were of the non-V600E type (Figure 1B).

### Clinicopathological Features of Patients With Double-Mutant CRC in Cohort Data Associated With TCGA, MSKCC, and DFCI Datasets

In total, 2,347 CRC samples were identified in the cohorts associated with TCGA, MSKCC, and DFCI datasets. The clinicopathological characteristics, MSI status, and TMB for patients with double-mutant CRC are summarized in Table 3. The clinicopathological features of patients with double-mutant CRC, namely age (average), sex, tumor site, histological type, tumor grade, and stage data, were obtained from the cBioPortal. MSI status and TMB were classified as described in materials and methods. However, some patients, for whom the data of histological type and MSI status were unavailable (indicated by N/A in Table 3), were omitted from the percentage calculation. Mutations were identified more in males, and the occurrence of tumor sites was slightly higher in the right side of the colon. Regarding the histopathological type, the conventional type was the most common; however, mucinous and poor differentiation were also observed. Histological grades G2 and G3 were observed in most cases, whereas G1 was absent. With respect to the MSI status, 52.4 and 46.6% of the cases were classified as MSS and MSI cases, respectively (MSI-I, MSI-H). TMB-low and TMB-high were observed in 39.1 and 60.1% of the cases, respectively. CIMP-low and -high accounted for 60% and 40% of the cases, respectively. Regarding *BRAF* classification, 22.2% of double-mutant CRC cases were class 1, 0% were class 2, 16.7% were class 3, and 61.1% were unknown.

**TABLE 3:** Clinicopathological information regarding *KRAS*/*BRAF* double-mutant CRC (n = 29).

Characteristics	Categories	TCGA (n = 6)	MSKCC (n = 17)	DFCI (n = 6)	Total
Age (average)	x	46–84 (71.7)	24–78 (50.8)	61–86 (71.8)	24–84 (59.5)
Sex (%)	Male/Female	6 (100)/0 (0)	12 (70.6)/5 (29.4)	4 (66.7)/2 (33.3)	22 (75.9)/7 (24.1)
Tumor site (%)	Left/Right	2 (33.3)/4 (66.7)	8 (47.1)/9 (52.9)	2 (33.3)/4 (66.7)	12 (41.4)/17 (58.6)
Histological type (%)	Conventional	5 (83.3)	3 (25.0)	N/A	8 (44.4)
	Conventional with mucinous	0 (0)	3 (25.0)	N/A	3 (16.7)
	Mucinous	1 (16.7)	2 (16.7)	N/A	3 (16.7)
	PDC	0 (0)	4 (33.3)	N/A	4 (22.2)
	N/A	0	5	6	11
histological characteristics (%)					
Tumor grade (%)	G1/G2/G3	0 (0)/3 (50)/3 (50)	0 (0)/7 (58.3)/5 (41.7)	0 (0)/5 (83.3)/1 (16.7)	0 (0)/15 (62.5)/9 (37.5)
	N/A	0	5	0	5
Stage (%)	Ⅰ/Ⅱ/Ⅲ/Ⅳ	1 (16.7)/4 (66.7)/1 (16.7)/0 (0)	0 (0)/5 (29.4)/5 (29.4)/7 (41.2)	2 (33.3)/3 (33.3)/1 (16.7)/0 (0)	3 (10.3)/12 (41.4)/7 (24.1)/7 (24.1)
MSI status (%)	MSS/MSI-I/MSI-H	2 (33.3)/0 (0)/4 (66.7)	10 (58.8)/2 (11.8)/5 (29.4)	3 (75)/0 (0)/2 (25)	12 (52.4)/2 (8.7)/9 (39.1)
	N/A	0	0	1	1
TMB (%)	TMB-low (<12 mut/Mb)	1 (16.7)	8 (47.1)	N/A	9 (39.1)
	TMB-high (≥12 Mb)	5 (83.3)	9 (52.9)	N/A	14 (60.9)
CIMP	CIMP-low/CIMP-high	N/A	N/A	3 (60)/2 (40)	3 (60)/2 (40)
	N/A	6	17	1	24
*BRAF* class					
			0	2	8 (22.2)
	2	0			0 (0)
	3	1	4	1	6 (16.7)
	unknown	6	13	3	22 (61.1)

Mut, mutation, PDC, poorly differentiated adenocarcinoma; MSS; microsatellite stability; MSI-I; Microsatellite instability-intermediate; MSI-H; Microsatellite instability-high; TMB, tumor mutation burden; CIMP, CpG island methylator phenotype.

### Comparison of Clinicopathological Features, MSI Status, and TMB Among Patients With KRAS-Mutant, BRAF-Mutant, and Double-Mutant CRC Based on Integrated Analysis of Information Available in TCGA, MSKCC, and DFCI Datasets

**FIGURE 2:**
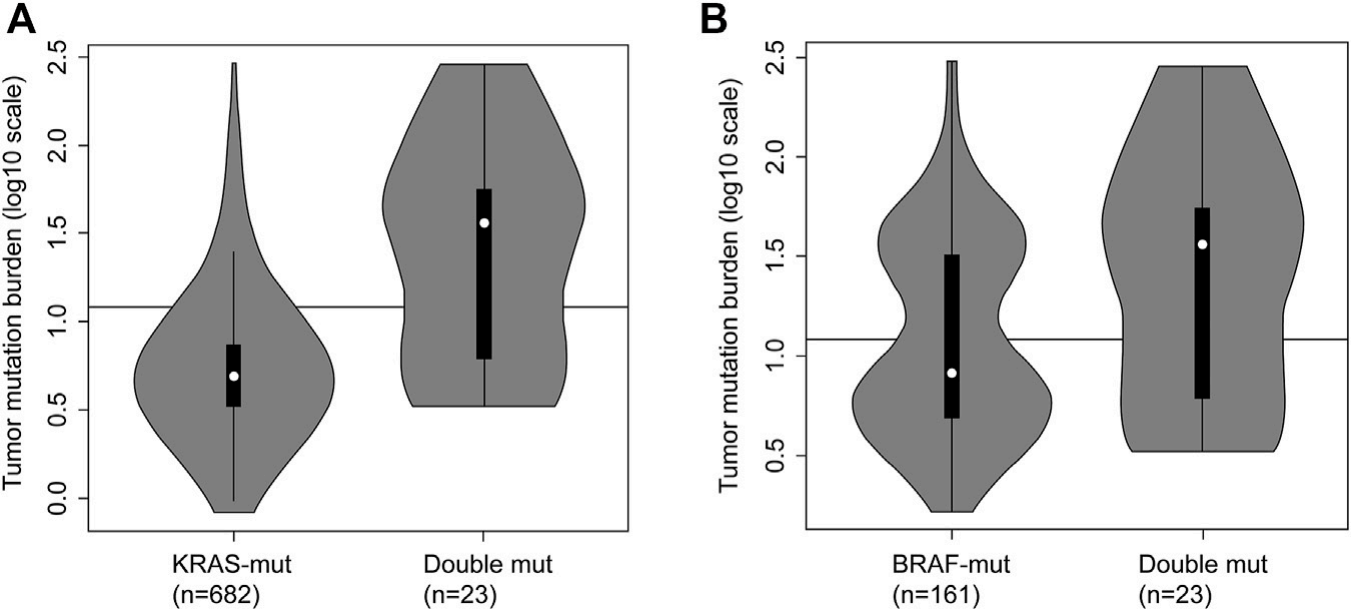
Tumor mutation burden (TMB) in *KRAS*-mutant CRC, *BRAF*-mutant CRC and double-mutant CRC. **(A)** TMB in *KRAS*-mutant CRC and double-mutant CRC. Comparison of TMB in patients with *KRAS*-mutant (n = 682) and double-mutant (n = 23) tumors based on integrated data hosted on The Cancer Genome Atlas (TCGA) and Memorial Sloan-Kettering Cancer Center (MSKCC; Mann–Whitney *U* test, *p* < 0.01). Black line indicating 12 mut/Mb represents the threshold for TMB-high. For *KRAS*-mutant CRC, the frequency of TMB-high was 9.2% (63/682); for double-mutant CRC, the frequency was 60.9% (14/23). **(B)** Tumor mutation burden in BRAF-mutant CRC and double-mutant CRC. Comparison of TMB in patients with BRAF-mutant (n = 161) and double-mutant (n = 23) tumors based on integrated data hosted on TCGA and MSKCC (the Mann–Whitney *U* test, *p* = 0.026). Black line indicating 12 mut/Mb represents the threshold for TMB-high. For BRAF-mutant CRC, the frequency of TMB-high was 42.9% (69/161); for double-mutant CRC, the frequency was 60.9% (14/23).

**FIGURE 3:**
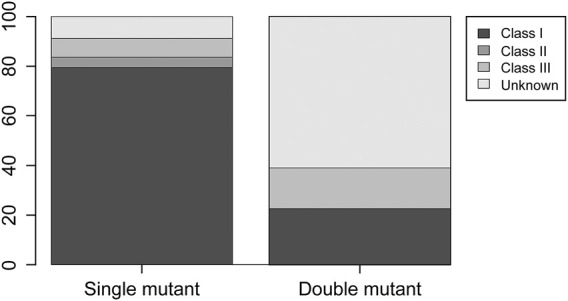
Distribution of *BRAF* class 1, 2, and 3 and unknown in *BRAF* single-mutant CRC and double-mutant CRC cases. Compared to single-mutant CRC, double-mutant CRC had significantly fewer *BRAF* class 1 and more unknown (*p* < 0.01).

The comparison among *KRAS*-mutant, *BRAF*-mutant, and double-mutant tumors in three datasets is summarized in Table 4. Double-mutant tumors were observed predominantly in males, and their frequency (75.9%) was significantly higher than that of the *KRAS*- and *BRAF*-mutant tumors (47.9 and 36.5%, respectively; *p <* 0.01) in males. Histological types of the double-mutant cases were significantly different from those observed in *KRAS*-mutant cases (*p =* 0.02); however, the difference between *BRAF*- and double-mutant cases was not significant (*p =* 0.59). Similarly, the histological grades differed significantly between *KRAS*- and double-mutant cases (*p <* 0.01), although not between *BRAF*- and double-mutant cases (*p* = 0.86). MSI status of double-mutant cases significantly differed from those observed in *KRAS*- and *BRAF*-mutant cases (*p <* 0.01 and *p =* 0.02, respectively). Contrarily, for CIMP, no significant difference among the three groups was observed. The mean TMB in *KRAS*-mutant CRC was 10.8 mut/Mb (median = 5.0), whereas that in *BRAF*-mutant CRC and double-mutant CRC was 24.7 mut/Mb (median = 8.2 mut/Mb) and 59.4 mut/Mb (median = 36.1), respectively. The TMB in double-mutant CRC was significantly higher than that in *KRAS*-mutant CRC (*p* < 0.01, Figure 2A) but was not significantly higher than that in *BRAF*-mutant CRC (*p* < 0.026, Figure 2B). TMB was frequently high in patients with double-mutant CRC compared to that in patients with *KRAS*-mutant tumors. However, the frequency of TMB did not differ among patients with *BRAF*- and double-mutant tumors (*p =* 0.026). Significantly fewer cases of double-mutant CRC were classified as *BRAF* class 1 and more were classified as unknown (*p* < 0.01) (Figure 3).

**TABLE 4:** Comparison of clinicopathological information among *KRAS*-mutant CRC; *BRAF*-mutant CRC; and double-mutant CRC obtained by integrating the information available in TCGA; MSKCC; and DFCI datasets (n = 2347).

Characteristics	Categories	*KRAS* mut (n = 849)	*BRAF* mut (n = 283)	Double mut (n = 29)	*p*-value (*KRAS* vs double)	*p*-value (*BRAF* vs double)
Frequency		36.2%	12.0%	1.2%		
Age (average)		20–93 (61.3)	26–90 (66.5)	24–86 (59.5)		
Sex (%)	Male	406 (47.9)	103 (36.5)	22 (75.9)	<0.01	<0.01
	Female	442 (52.1)	179 (63.5)	7 (24.1)		
	N/A	1	0	0		
Site (%)	Left	436 (53)	70 (25.3)	12 (41.4)	0.26	0.08
	Right	387 (47)	207 (74.7)	17 (58.6)		
	N/A	26	5	0		
Histological type (%)	Conventional	405 (74.6)	71 (56.3)	8 (44.4)	0.02	0.59
	Conventional with mucinous	51 (9.4)	17 (13.5)	3 (16.7)		
	Mucinous	52 (9.6)	23 (18.3)	3 (16.7)		
	PDC	32 (5.9)	13 (10.3)	4 (22.2)		
	Signet	2 (0.4)	0 (0)	0 (0)		
	MANEC	1 (0.2)	1 (0.8)	0 (0)		
	Medullary	0 (0)	1 (0.8)	0 (0)		
	N/A	139	35	5		
Grade (%)	G1	25 (3.7)	2 (0.9)	0 (0)	<0.01	0.86
	G2	554 (81.1)	146 (63.8)	15 (62.5)		
	G3	104 (15.2)	81 (35.4)	9 (37.5)		
	N/A	166	53	5		
Stage (%)	Ⅰ	88 (10.7)	37 (13.3)	3 (10.3)	0.07	0.88
	Ⅱ	174 (21.1)	96 (34.4)	12 (41.4)		
	Ⅲ	214 (26)	64 (22.9)	7 (24.1)		
	Ⅳ	347 (42.7)	82 (29.4)	7 (24.1)		
	N/A	26	3	0		
MSI status (%)	MSS	615 (90.7)	100 (62.5)	12 (52.2)	<0.01	0.02
	MSI-I	13 (1.9)	0 (0)	2 (8.7)		
	MSI-H	54 (7.9)	60 (37.5)	9 (39.1)		
	N/A	0	1	0		
CIMP	CIMP-low	117 (90.7)	29 (30.5)	3 (60)	0.08	0.32
	CIMP-high	12 (9.3)	66 (69.5)	2 (40)		
	N/A	38	26			
*BRAF* class						<0.01
	1	N/A	225 (79.5)	8 (22.2)		
	2	N/A	12 (4.2)	0 (0)		
	3	N/A	22 (7.8)	6 (16.7)		
	unknown	N/A	24 (8.5)	22 (61.1)		
					
TMB (mut/Mb) (%)	TMB-low	619 (90.8)	92 (57.1)	9 (39.1)	<0.01	0.026
	TMB-high	63 (9.2)	69 (42.9)	14 (60.9)		

Mut, mutation; PDC, poorly differentiated adenocarcinoma; MANEC, mixed adenoneuroendocrine carcinoma; MSS; microsatellite stability; MSI-I; Microsatellite instability-intermediate; MSI-H; Microsatellite instability-high; TMB, Tumor mutation burden. *p*-values were calculated by Fisher’s exact test.

## Discussion

In this study, the integrated results of three datasets from cBioPortal indicate that the frequency of the double-mutant CRC is 1.2%, which is greater than that of previous reports (0.02%) [13–19], This difference can be primarily attributed to the difference in sequencing methods used in the present versus previous studies. Most previous studies have reported the mutations only in hotspots, such as codons 12 and 13 of *KRAS* and codon 600 (i.e., V600E) of *BRAF*. However, in this study, we analyzed whole exome sequence (WES) and coding sequence (CDS) datasets of *KRAS* and *BRAF*. We inferred that the *KRAS* and *BRAF* mutations identified from the hotspots were mutually exclusive, as reported in several previous studies. The double mutations tended to occur at a relatively higher frequency outside hotspots (Figures 1A,B).

Double-mutant CRC demonstrated slightly higher occurrence in the right side of the colon and displayed mucinous differentiation and poor differentiation significantly more often than *KRAS*-mutant CRC. G3 was significantly more frequent than *KRAS*-mutant (Table 4). Double-mutant CRC demonstrated significantly more MSI-H than *KRAS*-mutant CRC (Table 4). Considering the clinicopathological features, several double-mutant CRCs differed significantly from *KRAS*-mutant CRC, although they displayed similar characteristics with *BRAF*-mutant CRC. Regarding TMB, double-mutant CRC demonstrated the highest TMB-high ratio, which significantly exceeded that of *KRAS*-mutant CRC (Table 4). These findings demonstrated that double-mutant CRC displayed a higher TMB value than those reported earlier [38]. To the best of our knowledge, this is the first study to analyze the three datasets collectively, identify the double mutations in CRC, and assess the clinicopathological features, MSI status, and TMB of KRAS/BRAF double-mutant CRC and compare them with *KRAS* and *BRAF* single mutation CRC.

Previous studies have identified the benefits of using EGFR inhibitors (i.e., cetuximab) for treating *KRAS-* and *BRAF*-mutant CRCs [39,40]. Tumor biology and drug sensitivity change with the site of the *KRAS* [41] and *BRAF* mutations. The drug sensitivity of non-V600 *BRAF* remains controversial, and there are several unclear points as discussed below [8]. Recent studies have reported that non-V600 *BRAF* mutations are associated with low response rates to EGFR inhibitors in CRC [42,43]. However, there have also been reports of patients with class 3 *BRAF* mutations who responded to EGFR inhibitors and chemotherapy [19]. In the current study, there were significantly more non-V600 *BRAF* mutations in the double-mutant CRC cases than in the single-mutant CRC cases. However, 61.1% of double-mutant CRC cases were classified as unknown. From these results, it appears that double-mutant CRC may have different biology compared to single-mutant CRC. Currently, no effective treatment has been established for double-mutant CRC. Attempts to treat double-mutant CRC by chemotherapy with FOLFOX (fluorouracil + folinic acid + oxaliplatin) have been presented in several case reports. Since the effect of EGFR could not be observed secondary to *KRAS* and *BRAF* mutations, all patients had received FOLFOX (fluorouracil + folinic acid + oxaliplatin) [21–25]. However, none of them exhibited a significant effect, and five of the seven patients died. From the results of this study, it can be observed that detection of double-mutant CRC is dependent on sequencing methods. As panel sequencing and whole-exome or -genome sequencing by next-generation sequencing has recently become widespread in clinical settings, double-mutant CRC may be detected more frequently. Further studies are necessary to modify and develop new chemotherapy regimens by including immune checkpoint inhibitors to achieve disease control in patients with *KRAS/BRAF* double-mutant CRC.

The study had certain limitations. First, the percentages calculated in this study might not be accurate, as we used different datasets, and the data for some of the tested characteristics were not available (N/A) or some categories had several instances of N/As. Second, the effect of data analysis methods that might incur false positives and false negatives and affect the overall frequency estimation was not evaluated in this study. Therefore, examining studies reporting the positive and negative false positives to gain insight into the influence of the data analysis methods in determining the frequency of double mutations could be interesting and useful. Third, we did not analyze the *NRAS*-mutant CRC, a biomarker for anti-EGFR treatment, in addition to *KRAS* and *BRAF* mutations. It has been presented that *NRAS* mutations are rare CRCs and do not appear to be associated with any of the molecular features, including mutation of *KRAS*, *BRAF*, *PIK3CA*, MSI, and CIMP [44]. Moreover, the frequency of double mutations involving *NRAS* mutations is rare [45,46]. Only three samples displayed triple mutations from the cases studied here (n = 2347), including *NRAS*, and analysis was impossible. Therefore, screening *NRAS* single and double mutants using a larger dataset might contribute to the development of an effective treatment strategy. Fourth, we could not carry out survival analysis because the stage and treatment methods for analyzing prognosis were not stringently standardized. Therefore, more data are required to determine whether the *KRAS/BRAF* double mutation can serve as a prognostic factor.

## Conclusion

We demonstrated that the occurrence frequency of the KRAS/BRAF double-mutant CRC was higher than that reported previously, suggesting that using a larger sample size and improved technologies that cover the sequencing information of WES and CDS datasets of cancer-related genes will be efficient in identifying the rare double mutations at a higher rate. Moreover, the findings suggest that double-mutant CRC is characterized by a higher occurrence in men and slight right-sided predominance. Pathologically, there were characterized by a significantly higher incidence of mucinous differentiation, poor differentiation, and a high histological grade (G3) than that of *KRAS*-mutant CRC. At the molecular level, significantly more MSI-high and higher TMB values were observed compared with those of *KRAS*-mutant CRC.

## Data Availability

The raw data supporting the conclusions of this article will be made available by the authors, without undue reservation.

## Supplementary Material

Supplementary Table S1 Summary of the legacy identifier (COSMIC), pathogenicity, and allele frequency of the KRAS and BRAF mutations of double-mutant CRC.

## Abbreviations

*BRAF,* v-RAF murine sarcoma viral oncogene homolog B1; CIMP, CpG island methylator phenotype; CDS, coding sequence; CRC, colorectal cancer; DFCI, Dana-Farber Cancer Institute; EGFR, epidermal growth factor receptor; KRAS, Kirsten rat sarcoma viral oncogene homolog; MSI, microsatellite instability; MSKCC, Memorial Sloan-Kettering Cancer Center; MSS, microsatellite stable; TCGA, The Cancer Genome Atlas; TMB, tumor mutation burden; WES, whole exome sequence.
